
JOURNAL INFORMATION
==============================
NLM Title Abbreviation: Chic Med J
NLM Journal ID: 0027576

The Chicago Medical Journal

Publisher: Biomedical Journal Digitization Project

ARTICLE INFORMATION
==============================
PMCID: PMC9750295
PMID: 37411222
Article version: 1
Subjects: Proceedings of Medical Societies

Annual Meeting of the Rock Island Medical Society

Print publication date: 1859 Jan
Volume: 16
Issue: 1
First page: 64
Last page: 64
License: This work is free of known copyright restrictions. For more information, please see PMC Back Issue Digitization.
License URL: https://creativecommons.org/publicdomain/mark/1.0/

==============================

panied by a sealed envelope, containing the name of the writer, and forwarded either to the chairman of the Committee on Prize Essays or to the permanent secretary of the society, on or before the first of May next. N. S. DAVIS,

*Permanent Secretary of the Society.*

MEDICAL SOCIETY.

We clip the following from the *Moline Independent:*

At the annual meeting of the Rock Island Medical Society the following officers were elected:

*President*—S. C. Plummer.
*Vice-President*—W. A. Knox.
*Recording Secretary*—C. Truesdale.
*Cor. Sec. and Treasurer*—E. H. Bowman.
*Board of Censors*—P. Gregg, W. A. Knox, W. F. Cady.

Dr. Bowman, the outgoing President, delivered a valedictory address, and Dr. Plummer read an interesting essay on the subject of "Puerperal Fever." Dr. Vincent of Hampton, and Dr. Hewitt of Camden were added to the society as members. The society meets again the second Wednesday in April.

FRESH VACCINE VIRUS,

**Constantly on hand and for Sale, by**

J. H. REED & CO., WHOLESALE DRUGGISTS, 144 & 146 LAKE STREET.

GALE BROTHERS,

APOTHECARIES AND CHEMISTS

**202 Randolph Street, Chicago.**

We are prepared to furnish Physicians with Medicines and Chemicals of the most reliable qualities, selected and prepared expressly for our own dispensing department.

New and rare Chemicals and Pharmaceutical Preparations furnished in quantities to suit Practitioners, to whom we recommend an examination of our stock.

ARTIFICIAL EYES!

We have a large assortment of *Artificial Human Eyes*, of the most approved manufacture. **GALE BROTHERS,**

APOTHECARIES AND PHARMACEUTISTS,

202 Randolph St., Chicago.
